# Identification and Analysis of Long Non-coding RNAs in *Leuciscus waleckii* Adapted to Highly Alkaline Conditions

**DOI:** 10.3389/fphys.2021.665268

**Published:** 2021-06-11

**Authors:** Xue Fei Zhao, Li Qun Liang, Hon Jung Liew, Yu Mei Chang, Bo Sun, Shuang Yi Wang, Bo Han Mi, Li Min Zhang

**Affiliations:** ^1^College of Wildlife and Protected Area, Northeast Forestry University, Harbin, China; ^2^Heilongjiang River Fisheries Research Institute, Chinese Academy of Fishery Sciences, Harbin, China; ^3^Higher Institution Center of Excellence (HICoE), Faculty of Fisheries and Food Science, Institute of Tropical Aquaculture and Fisheries, University of Malaysia Terengganu, Kuala Terengganu, Malaysia; ^4^College of Fisheries and Life Sciences, Shanghai Ocean University, Shanghai, China

**Keywords:** alkaline adaptation, China, Dali Lake, gene expression, high throughput sequencing, *Leuciscus waleckii*, lncRNA

## Abstract

*Leuciscus waleckii* is a freshwater fish that is known to inhabit the Dali Nor Lake, Inner Mongolia, China. The water in this lake has an HCO_3_^–^/CO_3_^2–^ concentration of 54 mM (pH 9.6) and a salinity of 0.6‰. The physiological mechanisms that allow this fish to tolerate these saline/alkaline conditions have yet to be elucidated. Transcriptional component analysis has shown that the expression levels of a large number of genes involved in the pathways responsible for osmo-ionoregulation and arachidonic acid metabolism pathway expression change significantly (*p* < 0.05) during the regulation of acid–base balance under high alkaline stress. In this study, we investigated the role of long non-coding RNAs (lncRNAs) during adaptation to high alkaline conditions. Fish were challenged to an NaHCO_3_-adjusted alkalinity of 0 mM, 30 mM (pH 9.44 ± 0.08), and 50 mM (pH 9.55 ± 0.06) for 20 days in the laboratory. Gill and kidney tissues were then collected for high-throughput sequencing assays. A total of 159 million clean reads were obtained by high-throughput sequencing, and 41,248 lncRNA transcripts were identified. Of these, the mean number of exons and the mean length of the lncRNA transcripts were 4.8 and 2,079 bp, respectively. Based on the analysis of differential lncRNA transcript expression, a total of 5,244 and 6,571 lncRNA transcripts were found to be differentially expressed in the gills and kidneys, respectively. Results derived from Gene Ontology (GO) and Kyoto Encyclopedia of Genes and Genomes (KEGG) analysis of the coding genes were correlated with the lncRNA expression profiles. GO analysis showed that many lncRNAs were enriched in the following processes: “transporter activity,” “response to stimulus,” and “binding.” KEGG analysis further revealed that metabolic pathways were significantly enriched. A random selection of 16 lncRNA transcripts was tested by RT-qPCR; these results were consistent with our sequencing results. We found that a large number of genes, with the same expression profiles as those with differentially expressed lncRNAs, were associated with the regulation of acid–base balance, ion transport, and the excretion of ammonia and nitrogen. Collectively, our data indicate that lncRNA-regulated gene expression plays an important role in the process of adaptation to high alkaline conditions in *L. waleckii*.

## Introduction

*Leuciscus waleckii* is an important farmed fish distributed in freshwater rivers and lakes in Russia, Mongolia, China, and Korea. The Dali Nor Lake located in Inner Mongolia, China, is a typical soda lake with an HCO_3_^–^/CO_3_^2–^ concentration of 54 mmol/L (54 mM, pH 9.6) and a salinity of 0.6‰. This lake is populated by *L. waleckii* because these fish have adapted to become tolerant to alkaline water. The population of *L. waleckii* in the Dali Nor Lake represents an important source of income for surrounding inhabitants and also an important source of food for birds migrating from Siberia to the south (Geng and Zhang, 1988; Zhang et al., 2008; Chi et al., 2010). Living in water that is highly alkaline requires specific physiological mechanisms to modulate basal metabolic needs. Although *L. waleckii* is an important fish that can live in such environments, the physiological mechanism underlying its tolerance to high alkaline conditions has yet to be elucidated.

To the best of our knowledge, research related to the mechanisms of alkaline adaptation in *L. waleckii* has hitherto focused on the screening and identification of functional genes involved in this adaptability. For example, Chen et al. (2019) sequenced the livers of *L. waleckii* before and after migration and spawning and successfully identified a large number of genes that are believed to be involved in acid–base regulation, nitrogenous waste excretion, and stress response; however, the precise molecular and functional mechanisms underlying these observations have yet to be investigated. In a previous study, Xu et al. (2017) investigated the evolutionary mechanisms of *L. waleckii* adapted to the extreme alkaline environment in the Dali Nor population and identified a set of selective genomic regions that showed key differences when compared between a population of *L. waleckii* from Dali Nor Lake and a freshwater population from River Ussuri; however, the authors failed to expand further on these spatial differences. More recently, Wang et al. (2021) investigated a large population of freshwater fish distributed around the same geographical location as the Dali Nor population. Eliminating differences in terms of habitat and other environmental conditions, these authors focused on comparing specific genomic regions between these populations. Sequencing results showed that the genomic regions under selective analysis were greatly reduced (Wang et al., 2021). However, pretranscriptional analysis identified a large number of differentially expressed genes that were related to salt/alkali adaptation (Xu et al., 2013a; Chang et al., 2014). Therefore, it is speculated that that there is only limited scope for the regulation of adaptability to environments with high alkalinity at the level of the genome. Previous work established that epigenetic regulation can help organisms respond quickly to stressful external environments without modifying any DNA sequences (Barghi et al., 2017). Therefore, it is suggested that the adaptation of *L. waleckii* in the Dali Nor Lake to high alkali conditions may be achieved at the epigenetic level.

Long non-coding RNAs (lncRNAs) are a class of epigenetic regulatory molecules with a total length of more than 200 nucleotides; these molecules can play an important role in the regulation of gene expression (Waseem et al., 2020). LncRNA was first identified in mice in 1989 (Brannan et al., 1990) but were initially perceived to represent transcriptional noise without a biological function (Mercer and Mattick, 2013). Brown et al. (1992) subsequently discovered that the Xist lncRNA was involved in the regulation of X chromosome inactivation in mammals. Since then, the attention of researchers has been drawn to non-coding RNA. Tens of thousands of lncRNAs have now been identified in humans, zebrafish, and mice (Zhao et al., 2016; Hu et al., 2018). LncRNAs can mediate the regulation of target genes by interacting with RNA, DNA, or proteins and are known to play a role in reproduction (Su et al., 2020), embryonic development (Kuang et al., 2020), sex differentiation (Cai et al., 2019), immunity (Liu et al., 2020), and metabolism (Li et al., 2020) at the transcriptional, posttranscriptional, and epigenetic levels. Other studies, involving fish, have shown that lncRNAs also play an important role in the network regulation of gene expression to cope with stress (Dettleff et al., 2020).

The kidneys and gills are the most important organs responsible for the homeostatic control of the internal osmotic milieu in euryhaline teleosts (Evans, 1979; Eddy, 1982). Gao et al. (2020) studied the histology of *L. waleckii*, and the results showed that on the 20th day, the number of chloride cells in the gills of Dali Nor *L. waleckii* at 30 and 50 mM alkalinity increased significantly compared with the control group. The chloride cells in the 50 mM alkalinity treatment were arranged and stacked more compactly, pavement cells became larger, and the cell surface thickened. In the freshwater population treated with 30 mM alkalinity, the number of chloride cells was significantly higher compared to that of the 50 mM group. However, under alkalinity stress, the gill fragments were damaged, and pavement cells, pillar cells, and blood cells were severely fused and fell off. Therefore, to further explore the potential role of lncRNAs in the process of high alkaline adaptation, we used 50 mM NaHCO_3_ to simulate the alkalinity conditions of Dali Nor Lake in our laboratory. As a comparison, we also created additional conditions in which the concentrations of NaHCO_3_ were 30 and 0 mM. Fish were exposed to these conditions for 20 days and then screened for differentially expressed lncRNAs. We sequenced tissues from the gills and kidneys of *L. waleckii* to identify lncRNAs that were closely associated with the regulation of high alkaline adaptation. Our overall goal was to provide a foundation for the future analysis of the molecular mechanisms that underlie adaptation.

## Materials and Methods

### Ethics Statement

All animal procedures were performed according to the Guidelines for Care and Use of Laboratory Animals of the Heilongjiang River Fisheries Research Institute of the Chinese Academy of Fishery Sciences.

### Animal and Alkalinity Stress Experiment

A total of 45 experimental fish with an average body weight of 22.51 ± 4.63 g were collected from an F4 generation after artificially propagating a population of fish extracted from the Dali Nor Lake. Fish were cultured in low alkalinity water at the Hulan Experimental Station of the Heilongjiang River Fisheries Research Institute (126.63°E, 45.97°N). Before the experiment, the fish were conditioned in a circulating controllable aquarium (42.6 cm L × 28.4 cm W × 29.3 cm H) for a week and fasted for 48 h before experimentation. Three parallel groups were prepared for each bicarbonate alkalinity gradient using NaHCO_3_ at 50 mM (pH 9.55 ± 0.06), 30 mM (pH 9.44 ± 0.08), and 0 mM (control) in triplicate. The control group received aerated tap water for 24 h. The basicity of the experiment was increased gradually at a rate of 5 mmol/L per day. In each aquarium, 50% of the total water volume was exchanged twice a day. Every day, a drop of methyl orange was added to the water and the bicarbonate concentration was titrated with 0.02 mmol/L HCl; this was repeated three times, and the average value was taken as the measure of alkalinity. Water quality was monitored with a YSI analyzer (Table 1). After 20 days of exposure, the gill and kidney tissues were collected, and five fish samples were pooled for sequencing assays from each group.

**TABLE 1:** Experimental water conditions.

	**0 mM**	**30 mM**	**50 mM**
T (°C)	19.55 ± 2.57	19.66 ± 1.19	19.40 ± 1.12
pH	7.51 ± 0.11	9.44 ± 0.08	9.55 ± 0.06
Dissolved oxygen (mg/L)	7.37 ± 0.39	7.54 ± 0.35	7.52 ± 0.34
Specific conductance (ms/cm)	0.12 ± 0.01	2.64 ± 0.10	4.23 ± 0.07
NH_4_^+^ concentration (mg/L)	2.49 ± 1.03	1.47 ± 0.42	1.35 ± 0.27
NH_3_ concentration (mg/L)	0.05 ± 0.05	1.13 ± 0.41	1.25 ± 0.33
Alkalinity (mmol/L)	0.05 ± 0.05	29.65 ± 0.96	49.69 ± 0.41

### RNA Quantification and Qualification

Total RNA was extracted using TRIzol reagent (Invitrogen, Carlsbad, CA, United States) in accordance with the manufacturer’s instructions (Rio et al., 2010). RNA degradation and contamination were monitored on 1% agarose gels. The qualified total RNA was further purified using a NanoPhotometer^®^ spectrophotometer (IMPLEN, CA, United States). RNA concentration was measured using a Qubit^®^ RNA Assay Kit in a Qubit^®^ 2.0 Fluorometer (Life Technologies, CA, United States). RNA integrity was assessed using the RNA Nano 6000 Assay Kit and a Bioanalyzer 2100 system (Agilent Technologies, CA, United States).

### Library Preparation for LncRNA Sequencing

Ribonucleic acid (3 μg) was used as input material for RNA sample preparations. Ribosomal RNA (rRNA) was depleted using an Epicentre Ribo-zero^TM^ rRNA Removal Kit (Epicentre, United States), and rRNA-free residue was cleaned by ethanol precipitation. Sequencing libraries were generated using the rRNA-depleted RNA by NEBNext^®^ Ultra^TM^ Directional RNA Library Prep Kit for Illumina^®^ (NEB, United States) in accordance with the manufacturer’s recommendations (Parkhomchuk et al., 2009; Mills et al., 2013). In brief, fragmentation was carried out using a divalent cation under elevated temperature in NEBNext First Strand Synthesis Reaction Buffer (5X). First-strand cDNA was then synthesized using a random hexamer primer and M-MuL V Reverse Transcriptase (RNaseH-). Second-strand cDNA synthesis was subsequently performed using DNA Polymerase I and RNase H. In the reaction buffer, dTTP was replaced by dUTP. Remaining overhangs were converted into blunt ends *via* exonuclease/polymerase activities. After adenylation of the 3’ ends of DNA fragments, a NEBNext Adaptor with a hairpin loop structure was used for ligation to prepare for hybridization. To preferentially select cDNA fragments of 150∼200 bp in length, the library fragments were purified using the AMPure XP system (Beckman Coulter, Beverly, United States). Then, 3 μl of USER Enzyme (NEB, United States) was used with size-selected and adaptor-ligated cDNA at 37°C for 15 min followed by 5 min at 95°C before PCR. The PCR was performed with Phusion High-Fidelity DNA polymerase, Universal PCR primers, and the Index (X) Primer. Finally, products were purified (AMPure XP system), and library quality was assessed on an Agilent Bioanalyzer 2100 system. Clustering of the index-coded samples was performed on a cBot Cluster Generation System using a TruSeq PE Cluster Kit v3-cBot-HS (Illumina) according to the manufacturer’s instructions. After cluster generation, the libraries were sequenced on an Illumina Hiseq 4000 platform (Novogene Bioinformatics Technology Corporation, Beijing, China), and 150-bp paired-end reads were generated.

### Transcriptome Assembly and LncRNA Identification

Clean data were obtained from the raw data by removing (i) reads containing adapter and ploy-N and (ii) low-quality reads as determined through Perl scripts. Then, the Q20, Q30, and GC content of the clean data were calculated. All downstream analyses were based on clean data of high quality. Reference genome and gene model annotation files were downloaded directly from the relevant genome website (NCBI Accession Number GCA_900092035.1) (Xu et al., 2017). An index of the reference genome was built using bowtie 2 v2.2.8, and paired-end clean reads were aligned with the reference genome using HISA T2 (Langmead and Salzberg, 2012) v2.0.4. The mapped reads for each sample were assembled by StringTie (v1.3.1) (Pertea et al., 2016) in a reference-based approach. StringTie uses a novel network flow algorithm as well as an optional *de novo* assembly step to assemble and quantify full-length transcripts representing multiple splice variants for each gene locus.

LncRNAs were filtered using a highly stringent filtering program. Firstly, a large number of single-exon transcripts with low expression levels and low credibility in the assembled transcripts were filtered; transcripts with more than 2 exon numbers were selected; and transcripts shorter than 200 bp were removed. Furthermore, transcripts that overlapped with the database annotation exon region were identified by Cuffcompare software, and the lncRNAs that overlapped with the spliced transcript exon region in the database were included in the follow-up analysis as database annotated lncRNAs. The expression levels of each transcript were calculated by Cuffquant, and transcripts with fragments per kilobase per million mapped reads (FPKM) ≥ 0.5 were selected. Coding-Non-Coding-Index (CNCI) (Sun et al., 2013), Coding Potential Calculator (CPC) (Kong et al., 2007), and Pfam-sca (Punta et al., 2012) were used to predict the coding potential of transcripts. Transcripts predicted with coding potential by either/all of the four tools above were filtered out, and those without coding potential were considered to be our candidate set of lncRNAs.

### Target Gene Prediction and Differential Expression Analysis

*Cis*-acting lncRNAs are known to target neighboring genes. Coding genes 10 kb/100 kb upstream and downstream of lncRNAs were identified, and their functions were analyzed. *Trans*-acting lncRNAs were identified by their expression levels (Derrien et al., 2012). To analyze function, bioinformatics methods were employed at the Novogene Bioinformatics Technology Corporation to calculate the correlations between lncRNA and coding gene expression following Cai et al. (2019). Cuffdiff (v2.1.1) was used to calculate FPKMs for the lncRNAs and coding genes in each sample (Trapnell et al., 2010). Gene FPKMs were computed by summating the FPKMs of transcripts in each gene group.

The Ballgown suite includes functions for the interactive exploration of transcriptome assembly, the visualization of transcript structures and feature-specific abundances for each locus, and the *post-hoc* annotation of assembled features to annotated features (Frazee et al., 2014). Cuffdiff provides statistical routines to determine differential expression in digital transcript or gene expression data using a model based on the negative binomial distribution (Trapnell et al., 2010). Transcripts with a *p* < 0.05 were classified as being differentially expressed.

Gene Ontology (GO) enrichment analyses of differentially expressed genes or lncRNA target genes were implemented using the GOseq package (Young et al., 2010) in R (R Core Team, 2014) to correct for gene length bias. GO terms with a corrected *p* < 0.05 were considered to be significantly enriched by differentially expressed genes. The Kyoto Encyclopedia of Genes and Genomes (KEGG) database is a resource for understanding high-level functions and utilities of the biological system from large-scale molecular datasets generated by high-throughput experimental technologies^1^ (Kanehisa et al., 2008). We used the KOBAS 3.0 platform^2^ to test the statistical significance of enrichment for the differentially expression genes or lncRNA target genes in KEGG pathways (Mao et al., 2005) with *p* < 0.05 considered significantly enriched pathways.

### RT-qPCR Validation and Statistical Analyses

To explore the validity of the RNA sequencing data, 16 differentially expressed transcripts of interest were selected for quantitative real-time PCR. The RNA samples for validation using RT-qPCR were the same as those used for Illumina sequencing. LnRNA-specific primers for validation and the temporal expression analysis of selected lncRNAs were designed using Primer3 Plus software^3^. The sequences of the specific primer sets are listed in Supplementary Table 1. Total RNA was extracted using Invitrogen Ambion TRIzol LS Reagent (Life Technologies Corp.). cDNA was synthesized by reverse transcription from 500 ng of total RNA using a PrimeScript RT Reagent Kit with gDNA Eraser (TaKaRa Bio Inc., China) according to the manufacturer’s instructions. Quantitative real-time PCR was performed using an SYBR PrimeScript qRT-PCR Kit (TaKaRa Bio Inc.) and an ABI 7500 Real-Time PCR System (Life Technologies Corp.). The QPCR conditions were as follows: predenaturation at 95°C for 30 s, 40 cycles of 95°C for 5 s, and 60°C for 34 s and one melting curve cycle at 95°C for 15 s, 60°C for 1 min, and 95°C for 15 s. All RT-qPCRs for each lncRNA were carried out in triplicate with three technical replicates per experiment. The cycle threshold (R. Core) values and raw fluorescence data were extracted using ABI 7500 software (Thermo Fisher Scientific Inc.). Baseline correction was performed with a baseline trend configured in terms of fluorescence data for 3–20 cycles. Background-corrected data were used to calculate PCR efficiency using LinRegPCR. The PCR efficiency for each individual sample was derived from the slope of the regression line fitted to a subset of baseline-corrected data points in the log-linear phase using the following equation: Efficacy (E) = 10^−1/*slope*^. Cycle threshold values of all genes were normalized with the Ct value of the 18S rRNA gene to obtain the ΔCt value. The fold change in the expression of every gene in different tissues was calculated based on the PCR efficiency and ΔCt values using the 2^–ΔΔCt^ method (Kenneth and Thomas, 2001).

## Results

### Sequencing Results

To further explore the role of lncRNAs in the process of high alkaline adaptation, we used an Illumina sequencing platform to sequence gill and kidney tissues following different alkalinity treatments at a depth of 10 × RNA-Seq. Sequencing produced a total of 1,611,147,224 raw reads and 1,590,439,792 clean reads; the mean percentage of clean reads was 98.72% (Table 2). The mean Q20 of each sample was 97.44%, and the mean Q30 was 92.99% (Table 2). A mean of 74.64% of clean reads were mapped to the reference genome of *L. waleckii* (NCBI Accession Number GCA_900092035.1), in which the mean proportions of multiple mapped and uniquely mapped reads were 2.26% and 72.39%, respectively (Table 2).

**TABLE 2:** Summary of transcriptomic sequencing results.

	**Gill**	**Kidney**
Total raw reads	791,291,488	819,855,736
Total clean reads	781,539,370	808,900,422
Total mapped	75.02	74.27
Average error rate (%)	0.03	0.03
Average Q20 (%)	97.41	97.48
Average Q30 (%)	92.90	93.08
Total mapped (%)	75.02	74.27
Average multiple mapped (%)	2.07	2.45
Average uniquely mapped (%)	72.95	71.82

### Identification of LncRNAs in the Gills and Kidneys of *L. waleckii*

**FIGURE 1:**
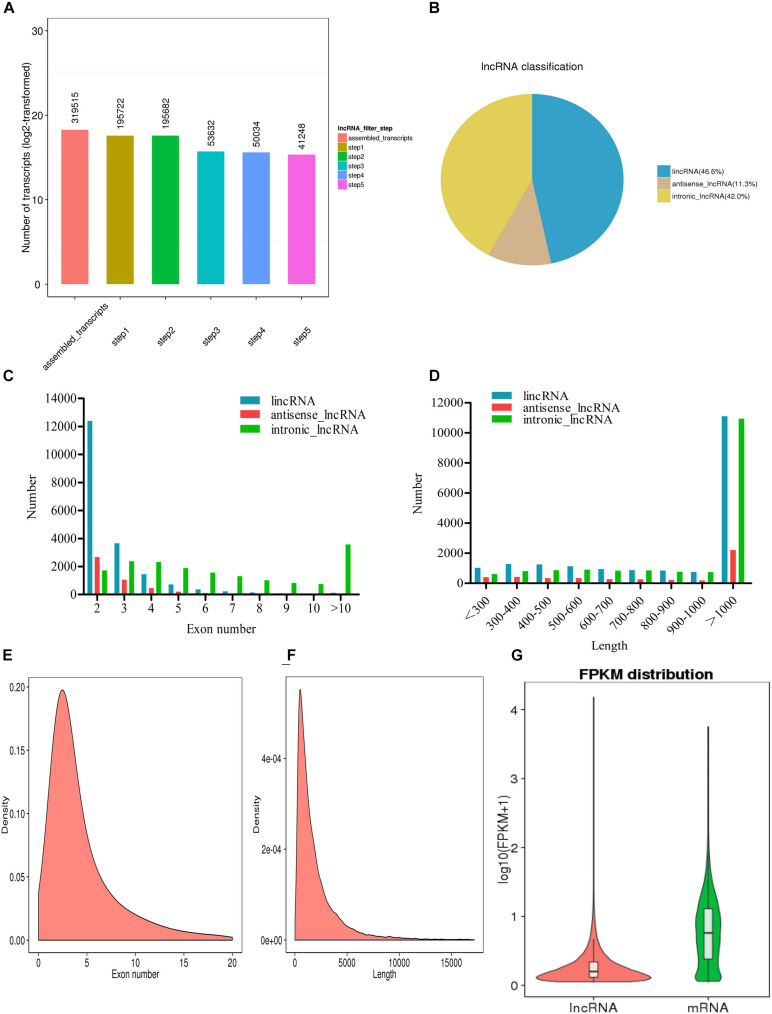
Long non-coding RNA (lncRNA) sequencing in *L. waleckii*. **(A)** The screening of lncRNA transcripts. **(B)** The distribution of lncRNA transcript types. **(C)** The distribution of exon number per lncRNA transcript type. **(D)** The distribution of exon lengths per lncRNA transcript type. **(E)** The distribution of exon numbers in lncRNA transcripts. **(F)** A length distribution map of lncRNA transcripts. **(G)** The expression levels of all lncRNA transcripts and mRNA transcripts.

A total of 319,515 transcripts were screened by exon number, transcript length, annotation, expression level, and coding power prediction, and a total of 41,248 potential lncRNA transcripts were obtained (Figure 1A). According to their position in the genome, and the closest distance of the protein coding genes, lncRNA transcripts can be divided into three types (Figure 1B): large intergenic non-coding RNAs (lincRNAs, 46.6% of the total); antisense lncRNAs (11.3%); and intronic lncRNAs (42%). The number of exons in the different types of lncRNA transcripts was compared; in most cases, there were 2–5 exons of lincRNAs and antisense lncRNAs. The distribution of intronic lncRNA exons was more uniform, with more than 10 in many cases (Figure 1C). The length distribution of these three types of lncRNA was similar with most being more than 1,000 nucleotides in length (Figure 1D).

In addition to the 41,248 non-coding lncRNA transcripts, Illumina RNA-seq produced 23,560 coding transcripts. Results showed that the mean number of exons was 4.8; this was lower than the 10.5 exons associated with coding transcripts (Figure 1E). The mean length of lncRNA transcripts was 2,079 bp; this was slightly lower than that of the coding transcripts (2,081 bp) (Figure 1F). We also found that coding transcripts with two and three exons accounted for 17.02% of all coding genes; this was much lower than the 57.90% of lncRNA transcripts (Supplementary Table 2). In addition, the average FPKM value of the lncRNA transcripts was significantly lower than that of the mRNA transcripts, thus indicating that the expression levels of lncRNA transcripts were relatively low (Figure 1G).

### Screening of Significantly Differentially Expressed LncRNAs

**FIGURE 2:**
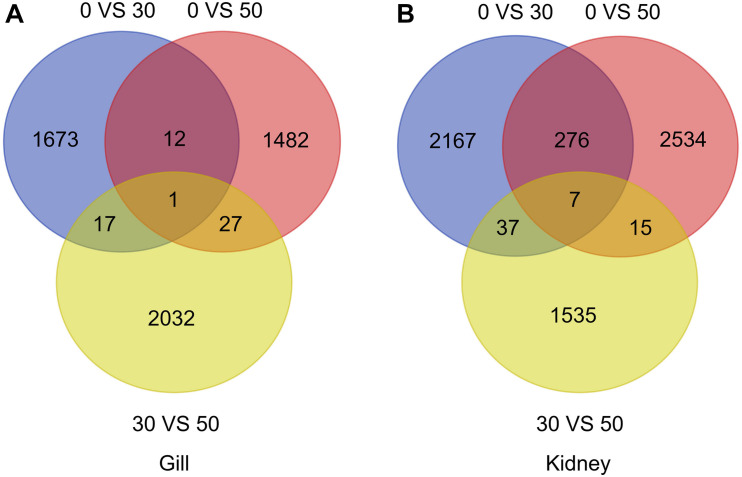
**(A)** Venn diagram of differentially expressed lncRNAs in gills. **(B)** Venn diagram of differentially expressed lncRNAs in kidneys of *L. waleckii* under different NaHCO_3_ concentrations.

Based on log2 > 2 or △FPKM > 2 as a screening condition for a significant difference in lncRNA expression, a total of 5,244 and 6,571 lncRNA transcripts were identified in the gills and kidneys, respectively. To further screen the lncRNA transcripts that were significantly related to the regulation of adaptability to high alkalinity in *L. waleckii*, Venn diagrams were constructed based on the lncRNAs that were expressed at significantly increased levels in the gill and kidney tissues when exposed to 0, 30, and 50 mM conditions. A total of 57 of these lncRNAs showed significant differential expression (*p* < 0.05) lncRNA transcripts were obtained among the three groups in gills (Figure 2A). Twenty lncRNAs showed an upward trend, seven showed a downward trend, and 30 showed a non-linear change with the alkalinity gradient. We also identified 335 lncRNA transcripts in the kidney that showed significant differential expression compared to the gills (*p* < 0.05; Figure 2B). Only 54 lncRNA transcripts showed an increase in expression with increasing alkalinity compared to 119 that showed a reduction in expression and 162 that changed expression but in a non-linear manner.

Only one lncRNA transcript (LNC_006608), found in the gills, was differentially expressed (*p* < 0.05) among the three groups; the expression pattern of LNC_006608 was related to that of the *ANO1* gene (anoctamin-1, log_2_FC_50/0_ = 4.58, calcium-activated chloride channel; Table 3). In contrast, a total of seven lncRNAs were identified in the kidney; of these, the expression profile of LNC_016538 was related to that of *ARRDC4* (arrestin domain-containing protein 4, log2FC_50/0_ = 4.52; a regulatory factor for glucose metabolism in mammals), while the expression profile of LNC_033457 was related to that of *RUFY2* (RUN, and FYVE domain-containing protein 2, log_2_FC_50/0_ = 3.56; a regulator of endocytosis) (Table 3).

**TABLE 3:** The differential expression of lncRNAs in the gills and kidneys of *L. waleckii* under different concentrations of NaHCO_3_.

**lncRNA_ID**	**Tissue**	**Number of coding genes related to lncRNA expression pattern**	** Uniport ID partial results**	**Partial description**
**lncRNAs showing significant differential expression across the three groups**				
LNC_006608	Gill	12	sp| Q5XXA6| ANO1_HUMAN	Anoctamin-1
LNC_016538	Kidney	35	sp| Q7Z5S9| TM144_HUMAN	Transmembrane protein 144
			sp| Q8NCT1| ARRD4_HUMAN	Arrestin domain-containing protein 4
LNC_033457	Kidney	12	sp| Q8WXA3| RUFY2_HUMAN	RUN and FYVE domain-containing protein 2
LNC_024078	Kidney	5	sp| Q01432| AMPD3_HUMAN	AMP deaminase 3
LNC_004201	Kidney	7	sp| Q9I8K7| CITE3_CHICK	Cbp/p300-interacting transactivator 3
LNC_011729	Kidney	5	sp| Q9Z273| TULP1_MOUSE	Tubby-related protein 1
LNC_038759	Kidney	1		
LNC_010544	Kidney	10	sp| Q9WV07| LOXE3_MOUSE	Hydroperoxide isomerase ALOXE3
**lncRNAs showing significant differential expression across the three groups**				
LNC_021054	Gill	7	sp| O88335| KCNJ1_MOUSE; sp| O02670| KCNJ5_PIG	ATP-sensitive inward rectifier potassium channel 1; G protein-activated inward rectifier potassium channel 4
LNC_033512	Gill	17	sp| Q9YH26| AT1A1_OREMO	Sodium/potassium-transporting ATPase subunit alpha-1
LNC_023391	Gill	6	sp| Q18PF5| RHCG1_TAKRU	Ammonium transporter Rh type C 1
LNC_013936	Gill	12	sp| Q99624| S38A3_HUMAN	Sodium-coupled neutral amino acid transporter 3
LNC_018373	Gill	8	sp| Q9Y696| CLIC4_HUMAN	Chloride intracellular channel protein 4
LNC_020745	Kidney	8	sp| P0DP35| CAM2B_XENLA	Calmodulin-2B
LNC_021122	Kidney	11	sp| Q4U116| S4A4_PIG	Electrogenic sodium bicarbonate cotransporter 1
LNC_032395	Kidney	13	sp| Q8CIW6| S26A6_MOUSE	Solute carrier family 26 member 6
LNC_016521	Kidney	538	sp| Q924C9| S26A3_RAT	Chloride anion exchanger
LNC_010650	Kidney	533	sp| Q9Y2D0| CAH5B_HUMAN	Carbonic anhydrase 5B
LNC_024081	Kidney	15	sp| P59158| S12A3_MOUSE	Solute carrier family 12 member 3
LNC_018956	Kidney	11	sp| Q805E9| ANF_OREMO; sp| Q9TT16| CLCN6_RABIT	Natriuretic peptides A; chloride transport protein 6

A total of 27 lncRNAs in the gills and 15 in the kidneys were found to be significantly differentially expressed (*p* < 0.05) when compared between the 0 and 50 mM groups and between the 30 and 50 mM groups, although no significant difference was detected when comparing the 0 and 30 mM groups. Of these, the KCNJ coding gene family (an ATP-sensitive inward rectifier potassium channel, log2FC_50/0_ = 4.59) was correlated to the expression profiles of LNC_021054 in the gills. These data indicated that *Kcnj1* (ATP-sensitive inward rectifier potassium channel 1) and *KCNJ5* (G protein-activated inward rectifier potassium channel 4) play important roles in potassium balance (Table 3). The *atp1a1* (sodium/potassium-transporting ATPase subunit alpha-1, log_2_FC_50/0_ = 2.43) coding gene was co-expressed with LNC_033512 in the gills and catalyzes the exchange of sodium and potassium ions across the plasma membrane (Table 3). The *CALM2-B* (calmodulin-2B, log_2_FC_50/0_ = 3.98) coding gene was co-located with LNC_020745 in the kidneys and belongs to the family of calmodulin genes (Table 3). Calmodulin can mediate Ca^2+^ to regulate a large number of proteins, including enzymes and ion channels. Furthermore, 12 and 276 lncRNAs were identified in the gills and kidney tissues, respectively, and shown to be significantly differentially expressed (*p* < 0.05) when comparing the 0 and 30 mM groups and the 0 and 50 mM groups, but not the 30 and 50 mM groups. Of these, the *RHCG1* coding gene (ammonium transporter Rh type C 1, log_2_FC_50/0_ = 4.03) was co-located with LNC_023391 in the gills to regulate ammonia secretion (Table 3). The *SLC4A4* coding gene (electrogenic sodium bicarbonate cotransporter 1, log_2_FC_50/0_ = 3.36) was co-located with LNC_021122 in the kidneys and is a sodium/bicarbonate co-transporter that regulates bicarbonate transport and intracellular pH (Table 3). The *SLC26A3* coding gene (chlorine anion exchanger) was co-expressed with LNC_016521 in the kidneys, while the *Slc26a6* coding gene (solute carrier family 26 member 6, log_2_FC_50/0_ = 2.62) was co-located with LNC_032395 in the kidneys; these have similar functions and encode an apical membrane anion exchange protein to maintain cell acid–base balance (Table 3). The *CA-VB* coding gene (carbonic anhydrase 5B) was co-expressed with LNC_010650 in the kidneys and regulates the reversible hydration of CO_2_ (Table 3). Significant lncRNA expression level differences were found between the 0 and 30 mM groups and between the 30 and 50 mM groups (*p* < 0.05), although there was no significant difference between the 0 and 50 mM groups that contained 17 and 37 lncRNAs in the gill and kidney tissues, respectively. Analysis of gill tissue identified the differential expression of LNC_013936; this lncRNA was co-located with *SLC38A3* (sodium-coupled neutral amino acid transporter 3, log_2_FC_50/30_ = 3.24), which mediates the co-transport of glutamine and sodium ions. LNC_018956 was co-located with the *NPPA* coding gene (natriuretic peptides A, log_2_FC_50/0_ = 15.11) and the *CLCN6* coding gene (chloride transport protein 6, log_2_FC_50/0_ = 15.11) in the kidneys and plays a key role in renal homeostasis and chloride transport (Table 3).

### Gills

#### Enrichment Analysis of Differentially Expressed LncRNAs: Co-located

**FIGURE 3:**
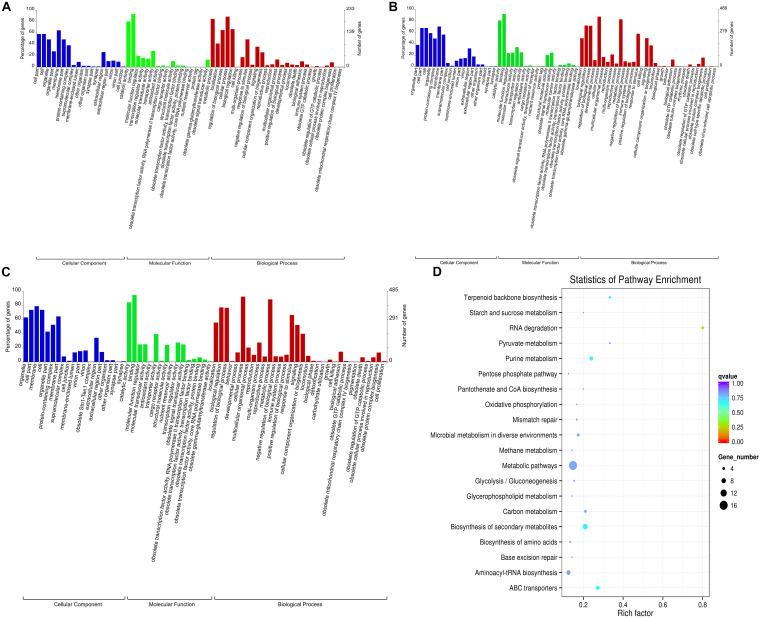
Gene Ontology (GO) and Kyoto Encyclopedia of Genes and Genomes (KEGG) enrichment maps of genes that were co-located with differentially expressed long non-coding RNAs (lncRNAs) in the gills of fish under different NaHCO_3_ concentrations. **(A)** GO enrichment map of the 0 and 30 mM groups. **(B)** GO enrichment map of the 0 and 50 mM groups. **(C)** GO enrichment map of the 30 and 50 mM groups. **(D)** KEGG enrichment map of the 30 and 50 mM groups.

Gene ontology enrichment analysis of coding genes in the gills that were 100 kb upstream and downstream of lncRNA transcripts found that 62 terms were enriched when the 0 and 30 mM groups were compared. Significant enrichment was observed for 18 terms in the cellular component (CC) category, 17 terms in the molecular function (MF) category, and 27 terms in the biological process (BP) category. When comparing the 0 and the 50 mM groups, and the 30 and 50 mM groups, we detected enrichment for 70 and 67 terms, respectively (*p* < 0. 05; Figures 3A–C). The three comparison groups all showed a greater number of terms that were enriched in the BP category, particularly with regard to biological regulation (GO:0065007), regulation of biological processes (GO:0050789), cellular processes (GO:0009987), immune system processes (GO:0002376), response to stimulus (GO:0050896), metabolic processes (GO:0008152), and other genes related to ion channel regulation, ion transport, stress response, immunity, and metabolism (Figures 3A–C). A large number of genes related to ion transport, transmembrane transport, and catalysis were also found to be significantly enriched in terms such as catalytic activity (GO:0003824), binding (GO:0005488), membranes (GO:0016020), transporter activity (GO:0005215), cell parts (GO:0044464), cells (GO:0005623), and structural molecule activity (GO:0005198); this was the case for both CC and MF (Figures 3A–C).

Kyoto encyclopedia of genes and genomes analysis of the same coding genes revealed that a large number of pathways were significantly enriched, including metabolic pathways, carbon metabolism pathways, ABC transporter signaling pathways, signaling pathways associated with the biosynthesis of amino acids, starch and sucrose metabolism, and other metabolic-related pathways (Figure 3D).

#### Enrichment Analysis of Differentially Expressed LncRNAs: Co-expressed

When predicting the potential target genes for the trans-regulatory action of lncRNA transcripts by expression level, fewer GO terms were enriched when comparing the 0 and 30 mM, 0 and 50 mM, and 30 and 50 mM groups when compared to co-located (Supplementary Figure 1). However, the terms that were enriched were similar to those in our co-located analysis. A number of terms were significantly enriched, including membrane-enclosed lumen (GO:0031974), transporter activity (GO:0005215), structural molecule activity (GO:0005198), signaling (GO:0023052), response to stimulus (GO:0050896), and biological adhesion (GO:0022610) (Supplementary Figure 1).

Kyoto encyclopedia of genes and genomes enrichment analysis of target genes showed that in addition to co-located enrichment, there was also significant enrichment in the pyruvate metabolism pathway, methane metabolism pathway, metabolic pathways, glycolysis/gluconeogenesis pathway, and other metabolic-related pathways (Supplementary Figure 1).

#### Kidneys

Analyzing the coding genes that were correlated to lncRNA expression patterns in the kidney, we found that the number of genes showing enriched GO terms was significantly higher than that of the gills. The range of terms showing enrichment was also significantly greater in the kidneys than in gills. However, the specific terms enriched by genes were similar when compared between the kidneys and the gills and were mainly related to ion transport and external stress, such as biological regulation (GO:0016747), transporter activity (GO:0005215), and response to stimulus (GO:0050896). In addition, many genes related to ion channels showed enrichment in synapse (GO:0045202), nutrient reservoir activity (GO:0045735), rhythmic process (GO:0048511), and cell proliferation (GO:0008283). The number of KEGG enrichment pathways of coding genes that were correlated to lncRNA expression patterns was slightly higher in the kidneys than in the gills, particularly with regard to various metabolic pathways (Supplementary Figure 2).

#### Verification of Differentially Expressed LncRNAs

**FIGURE 4:**
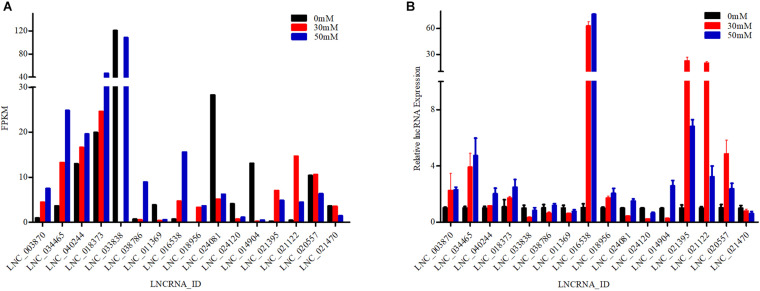
Validation of 16 differentially expressed long non-coding RNAs (lncRNAs) by RT-qPCR. Data represent the mean ± SEM. **(A)** Fragments per kilobase per million mapped reads (FPKM) value of lncRNAs in RNA-Seq. **(B)** RT-qPCR analysis of lncRNAs using RNA isolated from designated tissue fragments.

To explore the validity of RNA-seq, seven and nine lncRNA transcripts were selected from gill and kidney tissues, respectively. Therefore, a total of 16 differentially expressed lncRNA transcripts were analyzed by RT-qPCR in the gill and kidney tissues of the 0, 30, and 50 mM groups (Figure 4). Results showed that the trends in expression of the lncRNA transcripts detected by RT-qPCR were consistent with those derived from RNA-seq, thus confirming the validity of the RNA-seq data. RNA-seq and RT-qPCR results demonstrated a significant difference in the expression of lncRNA transcripts related to ion transport and ion channel regulation under high alkaline stress.

## Discussion

While there is a significant body of lncRNA research related to model organisms such as humans and mice, information relating to fish lncRNAs is relatively limited. Various studies have proven that lncRNAs can regulate gene expression and participate in a variety of physiological processes (Dey et al., 2014; Chu et al., 2020). Prior to this study, research on *L. waleckii* tended to focus on screening and identifying functional genes related to high alkaline tolerance (Chang et al., 2013, 2014; Xu et al., 2013a,b; Cui et al., 2015; Chen et al., 2019) but did not focus on the regulatory mechanisms responsible for differential gene expression in different tissues under high alkaline stress. In this study, we used a highly stringent filtering pipeline and the Illumina Hiseq 4000 platform to remove transcripts with protein-coding potential to minimize the selection of false-positive lncRNAs. Under 30 and 50 mM alkaline conditions, a total of 41,248 lncRNAs with multiple exons were identified in the gill and kidney tissues of *L. waleckii* (Figure 1A). Our results related to the identification of lncRNAs were highly reliable. Compared with protein-coding genes, putative lncRNA exons were identified to have fewer exons, shorter transcriptional lengths, and lower expression levels; these findings were consistent with the results of lncRNA sequencing in many different species (Moran et al., 2011; Ran et al., 2016). Differential expression analysis showed that the identified coding genes that were correlated with lncRNA expression patterns participated in many physiological processes, including ion regulation, acid–base regulation, ammonia transport, and metabolism. These are the first data to support the mechanisms by which gene expression is regulated by lncRNAs in the process of adapting to highly alkaline environments.

Since the use and interpretation of bioinformatics analysis based on RNA-Seq rely on known genes and functions, some important biological data may be overlooked, thus creating a potential limitation to this form of research (Khatri and Draghici, 2005; Tarca et al., 2013). However, as a promising high-throughput technology, RNA-Seq is still widely used in the mining of lncRNAs from aquatic animals and other species (Liz and Esteller, 2016; Zhan et al., 2016; Yue et al., 2018; Song et al., 2019). GO analysis not only identified enrichment in transport, the regulation of biological process, and response to stimuli; it also identified enrichment in potassium ion transport (GO:0006813), anion transport (GO:0006820), and sodium ion transport (GO:0006814), thus suggesting that lncRNAs are also involved in the regulation of gene expression related to ion transport. Metabolic process was significantly enriched in our KEGG analysis of *L. waleckii* gill and kidney tissues; this was consistent with previous transcriptomic studies (Chang et al., 2014). This finding indicates that metabolic pathways are widely involved in the adaptation to highly alkaline environments and that lncRNAs may play an important role in this process.

We compared the differential expression of lncRNA transcripts (*p* < 0.05) in the gills and kidneys of fish exposed to 0, 30, and 50 mM treatments and found that many coding genes that were correlated with lncRNA expression patterns were related to ion transport and the formation of ion channels. It is known that ion regulation plays a key role in maintaining internal homeostasis during the process of adaptation to high alkalinity, in which lncRNAs may also perform a similar regulatory role over the expression of related genes. We also found that the expression levels of lncRNA transcripts varied in a linear and a non-linear manner with alkalinity, thus suggesting that lncRNA transcripts are also involved in regulating the expression of target genes in different ways during adaptation to high alkalinity.

Analysis of differential expression in gill tissues showed that the *ANO1*-coding gene was related to the expression of LNC_006608; we found that *ANO1* was differentially expressed in all three groups (*p* < 0.05). *ANO1* is a calcium-activated chloride channel that is conserved in eukaryotes and expressed in all types of secretory epithelial tissues with anion selectivity and Cl^–^ regulatory functionality (Schroeder et al., 2008; Yang et al., 2008). The *chloride intracellular channel protein 4* (*CLIC4*)-coding gene (Table 3) co-located with LNC_018373 in the gills; this gene was selected for RT-qPCR verification and is highly conserved in vertebrates (Shorning et al., 2003). In mammals, the *CLIC4* protein can be automatically inserted into the cell membrane to form a membrane protein with chloride channel activity (Littler et al., 2005; Singh and Ashley, 2007). We found that the concentration of Cl^–^ in *L. waleckii* held in a 50 mM (high alkali) environment was significantly higher than that in a freshwater population of fish and higher than that in the control group. NaCl is the main electrolyte in body fluids, and regulating the concentration of Na^+^ and Cl^–^ is essential for osmotic regulation (Kaneko et al., 2008). Combined with the multiple lncRNAs related to Na^+^ regulation revealed in our results, this suggests that regulating the concentration of Na^+^ and Cl^–^ in the body plays an important role in maintaining the osmotic homeostasis of fish under a high NaHCO_3_ environment. Many members of the SLC4 and SLC26 gene families, which regulate acid–base balance, require Cl^–^ to participate in the transport of HCO_3_^–^. Therefore, the regulation of Cl^–^ homeostasis is closely related to acid–base regulation. Therefore, the expression levels of LNC_006608 and other lncRNA transcripts found in this study suggest an important role in the dynamic regulation of Cl^–^ in *L. waleckii* and allows them to live in the alkaline conditions that are present in Dali Nor Lake.

Endocytosis plays an important role in regulating the renewal and abundance of ion channels. Cells can transfer ion channel proteins located on the membrane into the cells *via* endocytosis and then degrade them *via* the lysosomes or recycle them back to the plasma membrane (Estadella et al., 2020). The *RUFY2*-coding gene was correlated with LNC_033457 expression patterns and is known to interact with Etk and participate in the regulation of endocytosis (Yang et al., 2002). LNC_033457 may play an important role in regulating the expression of ion channels on the plasma membrane. A significant amount of energy is needed to adapt to a highly alkaline environment. Interestingly, the *ARRDC4*-coding gene was correlated with the expression patterns of LNC_016538 and has been shown to inhibit glucose uptake (Patwari et al., 2009). With an increase in alkalinity, the transcriptional level of LNC_016538 increased gradually, thus suggesting that it may inhibit the expression of the *ARRDC4* gene or inhibit its physiological function, thus improving glucose uptake.

We identified a large number of differentially expressed lncRNA transcripts in the gills and kidneys when compared between the 0 and 30 mM, 0 and 50 mM, and 30 and 50 mM groups (*p* < 0.05). Of these, many lncRNA transcripts were not differentially expressed when compared between the 0 and 30 mM groups but were differentially expressed when compared between the other two groups (*p* < 0.05). Accordingly, we speculate that the expression level of lncRNA transcripts is specifically sensitive to a high alkaline environment. With increasing alkalinity, the transcription of certain lncRNAs changes significantly in order to regulate homeostasis. The *Kcnj1*- and *KCNJ5*-coding genes correlated with the expression patterns of LNC_021054 in the gills and are known to encode ATP-sensitive inward rectifier potassium channels in the kidneys, thus playing an important role in potassium homeostasis (Hebert and Wang, 1997; Mulatero et al., 2012). Sottejeau et al. (2010) previously found that the *atp1a1*-coding gene was co-expressed with LNC_033512 in the gills and was involved in the regulation of protein kinase C (PKC)-mediated NKA ion transport. With an increase in alkalinity, the lncRNA transcripts related to the expression pattern of genes related to potassium regulation were significantly differentially expressed (*p* < 0.05). In addition, the concentration of K^+^ is also believed to be closely related to osmotic regulation in fish (Martemyanov, 2020); as such, the expression of related lncRNA transcripts might play a role in the regulation of K^+^ dynamics under highly alkaline conditions.

The expression levels of some lncRNAs did not differ significantly when compared between the 30 and 50 mM groups; however, there was significant differential expression between the other two groups (*p* < 0.05). It is possible that these changes in expression may be related to the differences in alkalinity. It is possible that lncRNAs play an important role in the regulation of acid–base balance and ammonia metabolism. We found that the *SLC4A4*-, *SLC26A3*-, and *Slc26a6*-coding genes were related to the expression patterns of significantly differentially expressed lncRNAs. In a previous study, Chen et al. (2019) showed that several genes belonging to the *SLC4* gene family were differentially expressed in the livers of *L. waleckii* under highly alkaline conditions before and after migration. Chang et al. (2014) also found that three other genes (*SLC4A1*, *SLC4A4*, and *SLC4A2a*) were significantly upregulated in the gill and kidney tissues and that four members of the *SLC26* gene family (*SLC26a1*, *SLC26a5*, *SLC26a6*, and *SLC26A11*) were differentially expressed under highly alkaline conditions.

Acid–base regulation is one of the key regulatory processes underlying adaptation to highly alkaline environments and involves a series of regulatory processes related to ion levels. Anion exchangers of the *SLC4* and *SLC26* gene families are known to transport bicarbonate across the cell membrane (Alper and Sharma, 2013). Most members of the *SLC4* gene family encode Na^+^-independent Cl^–^ and HCO_3_^–^ exchangers (Alper, 2006). *SLC4A4* is widely expressed in various tissues involved in the regulation of ions, distributed on the basement membrane side of ionic cells in the gills, and responsible for Na^+^ and HCO_3_^–^ transportation (Romero et al., 1997; Hirata et al., 2003; Parks et al., 2007). In contrast, the *SLC26* gene family encodes a variety of anion exchangers and anion channels (Alper and Sharma, 2013). *SLC26a6* mainly mediates epithelial apical membrane Cl^–^/HCO_3_^–^ exchange; this represents one of the main pathways responsible for HCO_3_^–^ secretion (Shcheynikov et al., 2008). Both of these genes play an important role in intracellular acid–base balance and pH regulation (Boedtkjer and Aalkjaer, 2013; Romero et al., 2013). It is worth noting that a large number of lncRNAs have been annotated to *SLC26A3* and Cl^–^ and HCO_3_^–^ exchange activity in the kidneys (Melvin et al., 1999; Shcheynikov et al., 2006). However, the expression levels of these genes were significantly downregulated (*p* < 0.05), thus suggesting that the potential regulatory effect of lncRNAs on *SLC26A3* in the kidneys might be a key factor in manner by which *SLC26A3* can regulate ion balance. We also found that multiple lncRNA transcripts were correlated to the *CA-VB*-coding gene. The carbon anhydrase (CA) gene family plays an important role in CO_2_ emission and acid–base regulation in fish (Gilmour and Perry, 2009; Fehsenfeld and Weihrauch, 2013). *CA-VB* is widely distributed in the tissues of humans and mice and participates in carbon metabolism and pH homeostasis (Kasiliauskait et al., 2015). Previous studies have also found that *CA* and *CAHZ* genes are differentially expressed in populations of *L. waleckii* living in a highly alkaline environment (Xu et al., 2013b; Chen et al., 2019); these findings concur with our present data. This suggests that the *CA* gene family plays an important role in the regulation of acid–base balance in the body of *L. waleckii* and that lncRNA expression helps these important functional genes to perform their functions.

In addition, we found that LNC_023391 was differentially expressed and co-located to the *RHCG1* coding gene in the gills (*p* < 0.05). The NH_3_ channel rhesus glycoproteins (RH) can drive the Na^+^/H^+^ exchanger (NHE) to induce the efflux of NH_3_/NH_4_^+^ by producing H^+^ gradients, thus playing a universal role in the transport of ammonia (Weihrauch et al., 2004; Wright and Wood, 2009; Hwang et al., 2011). Fish living in alkaline water must be able to excrete ammonia and avoid endogenous ammonia toxication (Wilkie et al., 1994). A previous study of the ammonia excretion mechanism in *Nephelopsis obscura* exposed to different pH environments found that the mRNA expression levels of a Rhesus protein in the skin increased and induced ammonia transport (Quijada-Rodriguez et al., 2015). In another study, Chang et al. (2014) found that four ammonia transporters (*Rhag*, *Rhbg*, *Rhcg1*, and *Rhcg2*) were significantly upregulated in the gills of fish held in a highly alkaline environment; this finding concurs with our current results. Thus, *RHCG1* may be precisely regulated by lncRNA so as to avoid ammonia poisoning under highly alkaline conditions.

## Conclusion

In this study, many differentially expressed lncRNA transcripts were identified in the gill and kidney tissues of *L. waleckii* held in moderate and highly alkaline environments. A total of 5,244 lncRNA transcripts were identified in the gills and 6,571 in the kidneys. We also performed GO and KEGG analysis of the coding genes that were correlated to lncRNA expression patterns. GO analysis showed that many genes were enriched in a variety of terms, including “transporter activity,” “response to stimulus,” “binding,” “immune system process,” and “metabolic process.” KEGG results showed that metabolism-related pathways such as “metabolic pathways” and “carbon metabolism pathway” were significantly enriched. We suggest that lncRNAs may play an important metabolic role in the adaptation of *L. waleckii* to highly alkaline environments.

## Data Availability Statement

The datasets presented in this study can be found in online repositories. The names of the repository/repositories and accession number(s) can be found below: NCBI (accession: PRJNA722137).

## Ethics Statement

The animal study was reviewed and approved by The Laboratory Animal Management Committee Heilongjiang River Fisheries Research Institute, Chinese Academy of Fishery Sciences.

## Author Contributions

LL conceived the study. XZ, YC, BS, BM, and LZ performed the experimental research. XZ, BM, and SW analyzed the data. XZ and HL wrote the manuscript. All authors reviewed and approved the submitted version.

## Conflict of Interest

The authors declare that the research was conducted in the absence of any commercial or financial relationships that could be construed as a potential conflict of interest.

## Footnotes

1 http://www.genome.jp/kegg/

2 http://kobas.cbi.pku.edu.cn/

3 http://primer3plus.com/cgi-bin/dev/primer3plus.cgi

## References

[B1] Alper S. L. (2006). Molecular physiology of SLC4 anion exchangers. *Exp. Physiol.* 91 153–161. 10.1113/expphysiol.2005.031765 16239253

[B2] Alper S. L. Sharma A. K. (2013). The SLC26 gene family of anion transporters and channels. *Mol. Aspects Med.* 34 494–515. 10.1016/j.mam.2012.07.009 23506885 PMC3602804

[B3] Barghi S. Amiri M. Hajipour H. Namaki S. (2017). The effect of dietary constituents on regulation of epigenetic changes in cancer. *J. Babol Univ. Med. Sci.* 19 63–71.

[B4] Boedtkjer E. Aalkjaer C. (2013). Disturbed acid-base transport: an emerging cause of hypertension. *Front. Physiol.* 4:388. 10.3389/fphys.2013.00388 24399970 PMC3870919

[B5] Brannan C. I. Dees E. C. Ingram R. S. Tilghman S. M. (1990). The product of the H19 gene may function as an RNA. *Mol. Cell Biol.* 10 28–36. 10.1016/0166-6851(90)90215-8 1688465 PMC360709

[B6] Brown C. J. Hendrich B. D. Rupert J. L. Lafrenière R. G. Willard H. F. (1992). The human XIST gene: analysis of a 17 kb inactive X-specific RNA that contains conserved repeats and is highly localized within the nucleus. *Cell* 71 527–542. 10.1016/0092-8674(92)90520-M 1423611

[B7] Cai J. Li L. Song L. Y. Xie L. Luo F. Sun S. H. (2019). Effects of long term antiprogestine mifepristone (RU486) exposure on sexually dimorphic lncRNA expression and gonadal masculinization in Nile tilapia (*Oreochromis niloticus*). *Aquat. Toxicol.* 215:105289. 10.1016/j.aquatox.2019.105289 31491707

[B8] Chang Y. M. Tang R. Dou X. J. Tao R. Sun X. W. Liang L. Q. (2014). Transcriptome and expression profiling analysis of *Leuciscus waleckii*: an exploration of the alkali-adapted mechanisms of a freshwater teleost. *Mol. BioSyst.* 10 491–504. 10.1039/c3mb70318e 24382597

[B9] Chang Y. M. Tang R. Sun X. W. Liang L. Q. Chen J. P. Huang J. F. (2013). Genetic analysis of population differentiation and adaptation in *Leuciscus waleckii*. *Genetica* 141 417–429. 10.1007/s10709-013-9741-6 24154703

[B10] Chen B. H. Xu J. Cui J. Pu F. Peng W. Z. Chen L. (2019). Transcriptional differences provide insight into environmental acclimatization in wild amur ide (*Leuciscus waleckii*) during spawning migration from alkalized lake to freshwater river. *Genomics* 111 267–276. 30445216 10.1016/j.ygeno.2018.11.007

[B11] Chi B. J. Chang Y. M. Yan X. C. Cao D. C. Yong L. Gao Y. K. (2010). Genetic variability and genetic structure of *Leuciscus waleckii* Dybowski in Wusuli River and Dali Lake (in Chinese). *J. Fish. Sci. China* 17 228–235. 10.3724/SP.J.1011.2010.01351

[B12] Chu Q. Xu T. Zheng W. Chang R. Zhang L. (2020). Long noncoding RNA MARL regulates antiviral responses through suppression miR-122-dependent MAVS downregulation in lower vertebrates. *PLoS Pathog.* 16:e1008670. 10.1371/journal.ppat.1008670 32678830 PMC7390449

[B13] Cui J. Xu J. Zhang S. H. Wang K. Jiang Y. L. Shahid M. (2015). Transcriptional profiling reveals differential gene expression of Amur Ide (*Leuciscus waleckii*) during spawning migration. *Int. J. Mol. Sci.* 16 13959–13972. 10.3390/ijms160613959 26096003 PMC4490533

[B14] Derrien T. Johnson R. Bussotti G. Tanzer A. Guigó R. (2012). The GENCODE v7 catalog of human long noncoding RNAs: analysis of their gene structure, evolution, and expression. *Genome Res.* 22 1775–1789. 10.1101/gr.132159.111 22955988 PMC3431493

[B15] Dettleff P. Hormazabal E. Aedo J. Fuentes M. Valdes J. A. (2020). Identification and evaluation of long noncoding RNAs in response to handling stress in Red Cusk-Eel (*Genypterus chilensis*) via RNA-seq. *Mar biotechnol (New York, N.Y.)* 1 94–108. 10.1007/s10126-019-09934-6 31748906

[B16] Dey B. K. Mueller A. C. Dutta A. (2014). Long non-coding RNAs as emerging regulators of differentiation, development, and disease. *Transcription* 5:e944014. 10.4161/21541272.2014.944014 25483404 PMC4581346

[B17] Eddy F. B. (1982). Osmotic and ionic regulation in captive fish with particular reference to salmonds. *Comp. Biochem. Physiol. B Biochem. Mol. Biol.* 73B 125–141. 10.1016/0305-0491(82)90205-X

[B18] Estadella I. Pedrós-Gámez O. Colomer-Molera M. Bosch M. Sorkin A. Felipe A. (2020). Endocytosis: a turnover mechanism controlling ion channel function. *Cells* 9:1833. 10.3390/cells9081833 32759790 PMC7463639

[B19] Evans D. H. (1979). “Fish,” in *Comparative Physiology of Osmoregulation in Animals*, Vol. 1 ed. Maloiy G. M. P. (New York, NY: Academic Press), 305–390.

[B20] Fehsenfeld S. Weihrauch D. (2013). Differential acid–base regulation in various gills of the green crab *Carcinus maenas*: effects of elevated environmental pCO2. *Comp. Biochem. Phys. A Mol. Integr. Physiol..* 164 54–65. 10.1016/j.cbpa.2012.09.016 23022520

[B21] Frazee A. C. Pertea G. Jaffe A. E. Langmead B. Salzberg S. L. Leek J. T. (2014). Flexible analysis of transcriptome assemblies with ballgown. *bioRxiv* [Preprint] 10.1101/003665 bioRxiv: 003665,

[B22] Gao S. Chang Y. M. Zhao X. F. Sun B. Zhang L. M. Liang L. Q. (2020). The effect of different bicarbonate alkalinity on the gill structure of Amur Ide(*Leuciscus waleckii*) (in Chinese). *Acta Hydrobiol. Sin.* 44 736–743. 10.7541/2020.088 33132517 PMC7590850

[B23] Geng K. Zhang Z. (1988). Geomorphologic features and evolution of the Holocene lakes in Dalinor Area, the Inner Mongolia (in Chinese). *J. Beijing Normal Univ. (Nat. Sci.)* 4 94–101.

[B24] Gilmour K. M. Perry S. F. (2009). Carbonic anhydrase and acid-base regulation in fish. *J. Exp. Biol.* 212 1647–1661. 10.1242/jeb.029181 19448075

[B25] Hebert S. C. Wang W. H. (1997). Structure and function of the low conductance K(ATP) channel, ROMK. *Wien. Klin. Wochenschr.* 109 471–476. 10.1016/S0140-6736(05)63934-7 9261988

[B26] Hirata T. Kaneko T. Ono T. Nakazato T. Furukawa N. Hasegawa S. (2003). Mechanism of acid adaptation of a fish living in a pH 3.5 lake. *Am. J. Physiol.* 284 R1199–R1212. 10.1152/ajpregu.00267.2002 12531781

[B27] Hu X. Chen W. Li J. Huang S. L. Xu X. L. Zhang X. (2018). ZFLNC: a comprehensive and well-annotated database for zebrafish lncRNA. *Babol Univ. Med. Sci.* 19 63–71. 10.1093/database/bay114 30335154 PMC6193196

[B28] Hwang P. P. Lee T. H. Lin L. Y. (2011). Ion regulation in fish gills: recent progress in the cellular and molecular mechanisms. *Am. J. Physiol. Regul. Integr. Comp. Physiol.* 301 R28–R47. 10.1152/ajpregu.00047.2011 21451143

[B29] Kanehisa M. Araki M. Goto S. Hattori M. Hirakawa M. Itoh M. (2008). KEGG for linking genomes to life and the environment. *Nucleic Acids Res.* 36(suppl 1) D480–D484. 10.1093/nar/gkm882 18077471 PMC2238879

[B30] Kaneko T. Watanabe S. Lee K. (2008). Functional morphology of mitochondrion-rich cells in euryhaline and stenohaline teleosts. *Aqua Biosci. Monogr.* 1 1–62. 10.5047/absm.2008.00101.0001

[B31] Kasiliauskait A. Asait V. Juozapaitien V. Zubrien A. Matulis D. (2015). Thermodynamic characterization of human carbonic anhydrase VB stability and intrinsic binding of compounds. *J. Therm. Anal. Calorim.* 123 1–10. 10.1007/s10973-015-5073-3

[B32] Kenneth J. L. Thomas D. S. (2001). Analysis of relative gene expression data using real-time quantitative PCR and the 2-ΔΔ C T method. *Methods* 25 402–408. 10.1006/meth.2001 11846609

[B33] Khatri P. Draghici S. (2005). Ontological analysis of gene expression data: current tools, limitations, and open problems. *Bioinformatics* 21 3587–3595. 10.1093/bioinformatics/bti565 15994189 PMC2435250

[B34] Kong L. Zhang Y. Ye Z. Q. Liu X. Q. Zhao S. Q. Ping W. L. (2007). CPC: assess the protein-coding potential of transcripts using sequence features and support vector machine. *Nucleic Acids Res.* 35 345–349. 10.1093/nar/gkm391 17631615 PMC1933232

[B35] Kuang L. D. Lei M. Li C. Y. Guo Z. Q. Ren Y. J. Zhang X. Y. (2020). Whole transcriptome sequencing reveals that non-coding RNAs are related to embryo morphogenesis and development in rabbits. *Genomics* 112 2203–2212. 10.1016/j.ygeno.2019.12.016 31881265

[B36] Langmead B. Salzberg S. L. (2012). Fast gapped-read alignment with Bowtie 2. *Nat. Methods* 9 357–359. 10.1038/nmeth.1923 22388286 PMC3322381

[B37] Li D. Y. Wu L. H. Knox B. Chen S. Ning B. T. (2020). Long noncoding RNA LINC00844-mediated molecular network regulates expression of drug metabolizing enzymes and nuclear receptors in human liver cells. *Arch. Toxikol.* 94(Suppl. 2) 1637–1653. 10.1007/s00204-020-02706-5 32222775 PMC7773225

[B38] Littler D. R. Assaad N. N. Harrop S. J. Brown L. J. Pankhurst G. J. Luciani P. (2005). Crystal structure of the soluble form of the redox-regulated chloride ion channel protein CLIC4. *FEBS J.* 272 4996–5007. 10.1111/j.1742-4658.2005.04909.x 16176272

[B39] Liu W. Wang Z. Q. Liu L. Yang Z. H. Cao X. T. (2020). LncRNA Malat1 inhibition of TDP43 cleavage suppresses IRF3-initiated antiviral innate immunity. *Proc. Natl. Acad. Sci. U.S.A.* 117 23695–23706. 10.1073/pnas.2003932117 32907941 PMC7519350

[B40] Liz J. Esteller M. (2016). lncRNAs and microRNAs with a role in cancer development. *Biochim. Biophys. Acta* 1859 169–176. 10.1016/j.bbagrm.2015.06.015 26149773

[B41] Mao X. Z. Cai T. Olyarchuk J. G. Wei L. P. (2005). Automated genome annotation and pathway identification using the KEGG Orthology (KO) as a controlled vocabulary. *Bioinformatics* 21 3787–3793. 10.2307/1592215 15817693

[B42] Martemyanov V. I. (2020). Indicators of osmotic and ion regulation in the fish of the white sea. *J. Ichthyol.* 60 305–314. 10.1134/S0032945220020101

[B43] Melvin J. E. Park K. Richardson L. Schultheis P. J. Shull G. E. (1999). Mouse down-regulated in adenoma (DRA) is an intestinal Cl^–^/HCO_3_^–^ exchanger and is up-regulated in colon of mice lacking the NHE3 Na^+^/H^+^ exchanger. *J. Biol. Chem.* 274 22855–22861. 10.1074/jbc.274.32.22855 10428871

[B44] Mercer T. R. Mattick J. S. (2013). Structure and function of long noncoding RNAs in epigenetic regulation. *Nat. Struct. Mol. Biol.* 20 300–307. 10.1038/nsmb.2480 23463315

[B45] Mills J. D. Kawahara Y. Janitz M. (2013). Strand-specific RNA-Seq provides greater resolution of transcriptome profiling. *Curr. Genomics* 14 173–181. 10.2174/1389202911314030003 24179440 PMC3664467

[B46] Moran N. C. Cole T. Loyal G. Magdalena K. Barbara T.-V. Aviv R. (2011). Integrative annotation of human large intergenic noncoding RNAs reveals global properties and specific subclasses. *Gene Dev.* 25 1915–1927. 10.1101/gad.17446611 21890647 PMC3185964

[B47] Mulatero P. Tauber P. Zennaro M.-C. Monticone S. Lang K. Beuschlein F. (2012). KCNJ5 mutations in European families with nonglucocorticoid remediable familial hyperaldosteronism. *Hypertension* 59 235–240. 10.1161/hypertensionaha.111.183996 22203740

[B48] Parkhomchuk D. Borodina T. Amstislavskiy V. Banaru M. Hallen L. Krobitsch S. (2009). Transcriptome analysis by strand-specific sequencing of complementary DNA. *Nucleic Acids Res.* 37 e123. 10.1093/nar/gkp596 19620212 PMC2764448

[B49] Parks S. K. Tresguerres M. Goss G. G. (2007). Interactions between Na^+^ channels and Na^+^-HCO_3_^–^ cotransporters in the freshwater fish gill MR cell: a model for transepithelial Na^+^ uptake. *Am. J. Physiol. Cell Physiol.* 292 C935. 10.1152/ajpcell.00604.2005 17005600

[B50] Patwari P. Chutkow W. A. Cummings K. Verstraeten V. L. R. M. Lammerding J. Schreiter E. R. (2009). Thioredoxin-independent regulation of metabolism by the α-arrestin proteins. *J. Biol. Chem.* 284 24996–25003. 10.1074/jbc.m109.018093 19605364 PMC2757204

[B51] Pertea M. Kim D. Pertea G. M. Leek J. T. Salzberg S. L. (2016). Transcript-level expression analysis of RNA-seq experiments with HISAT, StringTie and ballgown. *Nat. Protoc.* 11 1650–1667. 10.1038/nprot.2016.095 27560171 PMC5032908

[B52] Punta M. Coggill P. C. Eberhardt R. Y. Mistry J. Tate J. Boursnell C. (2012). The Pfam protein families database. *Nucleic Acids Res.* 40 290–D301. 10.1093/nar/gkr1065 22127870 PMC3245129

[B53] Quijada-Rodriguez A. R. Treberg J. R. Weihrauch D. (2015). Mechanism of ammonia excretion in the freshwater leech *Nephelopsis obscura*: characterization of a primitive Rh protein and effects of high environmental ammonia. *Am. J. Physiol. Regul. Integr. Comp. Physiol..* 309 R692–R705. 10.1152/ajpregu.00482.2014 26180186 PMC4591366

[B54] R Core Team (2014). R: a language and environment for statistical computing. *Computing* 14 12–21.

[B55] Ran M. Chen B. Li Z. Wu M. Liu X. He C. (2016). Systematic identification of long non-coding RNAs in immature and mature porcine testes. *Biol. Reprod.* 94:77. 10.1095/biolreprod.115.136911 26935596

[B56] Rio D. C. Ares M. Hannon G. J. Nilsen T. W. (2010). Purification of RNA using TRIzol (TRI reagent). *Cold Spring Harb. Protoc.* 2010:db.rot5439. 10.1101/pdb.prot5439 20516177

[B57] Romero M. F. Chen A. P. Parker M. D. Boron W. F. (2013). The SLC4 family of bicarbonate (HCO_3_^–^) transporters. *Mol. Aspects Med.* 34 159–182. 10.1016/j.mam.2012.10.008 23506864 PMC3605756

[B58] Romero M. F. Hediger M. A. Boulpaep E. L. Boron W. F. (1997). Expression cloning and characterization of a renal electrogenic Na^+^/HCO_3_^–^ cotransporter. *Nature* 387 409–413. 10.1038/387409a0 9163427

[B59] Schroeder B. C. Cheng T. Jan Y. N. Jan L. Y. (2008). Expression cloning of TMEM16A as a calcium-activated chloride channel subunit. *Cell* 134 1019–1029. 10.1016/j.cell.2008.09.003 18805094 PMC2651354

[B60] Shcheynikov N. Wang Y. Park M. Ko S. Dorwart M. Naruse S. (2006). Coupling modes and stoichiometry of Cl^–^/HCO_3_^–^ exchange by slc26a3 and slc26a6. *J. Gen. Physiol.* 127 511–524. 10.1085/jgp.200509392 16606687 PMC2151520

[B61] Shcheynikov N. Yang D. K. Wang Y. Zeng W. Z. Karniski L. P. So I. (2008). The Slc26a4 transporter functions as an electroneutral Cl^–^/I^–^/HCO_3_^–^ exchanger: role of Slc26a4 and Slc26a6 in I^–^ and HCO_3_^–^ secretion and in regulation of CFTR in the parotid duct. *J. Physiol.* 586 3813–3824. 10.1113/jphysiol.2008.154468 18565999 PMC2538934

[B62] Shorning B. Wilson D. Meehan R. Ashley R. (2003). Molecular cloning and developmental expression of two Chloride Intracellular Channel (CLIC) genes in *Xenopus laevis*. *Dev. Genes Evol.* 213 514–518. 10.1007/s00427-003-0356-2 13680226

[B63] Singh H. Ashley R. H. (2007). CLIC4 (p64H1) and its putative transmembrane domain form poorly selective, redox-regulated ion channels. *Mol. Membr. Biol.* 24 41–52. 10.1080/09687860600927907 17453412

[B64] Song F. Wang L. Zhu W. Dong Z. (2019). Long noncoding RNA and mRNA expression profiles following igf3 knockdown in common carp, Cyprinus carpio. *Sci. Data* 6:190024. 10.1038/sdata.2019.24 30778253 PMC6380219

[B65] Sottejeau Y. Belliard A. Duran M. J. Pressley T. A. Pierre S. V. (2010). Critical role of the isoform-specific region in alpha1-Na,K-ATPase trafficking and protein Kinase C-dependent regulation. *Biochemistry* 49:3602. 20302352 10.1021/bi9021999 PMC4303032

[B66] Su T. Yu H. L. Luo G. Wang M. X. Xu D. Q. (2020). The Interaction of lncRNA XLOC-2222497, AKR1C1, and progesterone in porcine endometrium and pregnancy. *Int. J. Mol. Sci.* 21:3232. 10.3390/ijms21093232 32370225 PMC7247569

[B67] Sun L. Luo H. T. Bu D. C. Zhao G. G. Yu K. T. Zhang C. H. (2013). Utilizing sequence intrinsic composition to classify protein-coding and long non-coding transcripts. *Nucleic Acids Res.* 41:e166. 10.1093/nar/gkt646 23892401 PMC3783192

[B68] Tarca A. L. Bhatti G. Romero R. (2013). A comparison of gene set analysis methods in terms of sensitivity, prioritization and specificity. *PLoS One* 8:e79217. 10.1371/journal.pone.0079217 24260172 PMC3829842

[B69] Trapnell C. Williams B. A. Pertea G. Mortazavi A. Kwan G. van Baren M. J. (2010). Transcript assembly and quantification by RNA-Seq reveals unannotated transcripts and isoform switching during cell differentiation. *Nat. Biotechnol.* 28 511–515. 10.1038/nbt.1621 20436464 PMC3146043

[B70] Wang S. Y. Kuang Y. Y. Liang L. Q. Sun B. Zhao X. F. Zhang L. M. (2021). Resequencing and SNP discovery of Amur ide (*Leuciscus waleckii*) provides insights into local adaptations to extreme environments. *Sci. Rep.* 11:5064. 10.1038/s41598-021-84652-5 33658614 PMC7930030

[B71] Waseem M. Liu Y. L. Xia R. (2020). Long non-coding RNAs, the dark matter: an emerging regulatory component in plants. *Int. J. Mol. Sci.* 22:86. 10.3390/ijms22010086 33374835 PMC7795044

[B72] Weihrauch D. Steve M. David W. T. (2004). Ammonia excretion in aquatic and terrestrial crabs. *J. Exp. Biol.* 207 4491–4504. 10.1242/jeb.01308 15579545

[B73] Wilkie M. P. Wright P. A. Iwama G. K. Wood C. M. (1994). The physiological adaptations of the *Lahontan cutthroat trout* (*Oncorhynchus clarki henshawi*) following transfer from well water to the highly alkaline waters of Pyramid Lake, Nevada (pH 9.4). *Physiol. Zool.* 67 355–380.

[B74] Wright P. A. Wood C. M. (2009). A new paradigm for ammonia excretion in aquatic animals: role of rhesus (RH) glycoproteins. *J. Exp. Biol.* 212(Pt. 15) 2303–2312. 10.1242/jeb.023085 19617422

[B75] Xu J. Ji P. Wang B. Zhao L. Wang J. Zhao Z. (2013a). Transcriptome sequencing and analysis of wild Amur Ide (*Leuciscus waleckii*) inhabiting an extreme Alkaline-Saline Lake reveals insights into stress adaptation. *PLoS One* 8:e59703. 10.1371/journal.pone.0059703 23573207 PMC3613414

[B76] Xu J. Li J. T. Jiang Y. L. Peng W. Z. Yao Z. L. Chen B. H. (2017). Genomic basis of adaptive evolution: the survival of Amur Ide (*Leuciscus waleckii*) in an extremely alkaline environment. *Mol. Biol. Evol.* 34 145–159. 10.1093/molbev/msw230 28007977 PMC5854124

[B77] Xu J. Li Q. Xu L. M. Wang S. L. Jiang Y. L. Zhao Z. X. (2013b). Gene expression changes leading extreme alkaline tolerance in Amur ide (*Leuciscus waleckii*) inhabiting soda lake. *BMC Genomics* 14:682. 10.1186/1471-2164-14-682 24094069 PMC3852516

[B78] Yang J. Kim O. Wu J. Qiu Y. (2002). Interaction between tyrosine kinase Etk and a run domain- and FYVE domain-containing protein RUFY1 a possible role of ETK in regulation of vesicle trafficking. *J. Biol. Chem.* 277 30219–30226. 10.1074/jbc.M111933200 11877430

[B79] Yang Y. D. Cho H. Koo J. Y. Tak M. H. Cho Y. Shim W.-S. (2008). TMEM16A confers receptor-activated calcium-dependent chloride conductance. *Nature* 455 1210–1215. 10.1038/nature07313 18724360

[B80] Young M. D. Wakefield M. J. Smyth G. K. Oshlack A. (2010). Gene ontology analysis for RNA-seq: accounting for selection bias. *Genome Biol.* 11:R14. 10.1186/gb-2010-11-2-r14 20132535 PMC2872874

[B81] Yue C. Li Q. Yu H. (2018). Gonad transcriptome analysis of the Pacific oyster *Crassostrea gigas* identifies potential genes regulating the sex determination and differentiation process. *Mar. Biotechnol.* 20 206–219. 10.1007/s10126-018-9798-4 29516375

[B82] Zhan S. Dong Y. Zhao W. Guo J. Zhong T. Wang L. (2016). Genome-wide identification and characterization of long non-coding RNAs in developmental skeletal muscle of fetal goat. *BMC Genomics* 17:666. 10.1186/s12864-016-3009-3 27550073 PMC4994410

[B83] Zhang Y. Liang L. Q. Jiang P. Yu D. (2008). Genome evolution trend of common carp (*Cyprinus carpio L.*) as revealed by the analysis of microsatellite loci in a gynogentic family. *J. Genet. Genomics.* 35 97–103. 10.1016/S1673-8527(08)60015-6 18407057

[B84] Zhao Y. Li H. Fang S. S. Kang Y. Wu W. Hao Y. J. (2016). NONCODE 2016: an informative and valuable data source of long non-coding RNAs. *Nucleic Acids Res.* 44 D203–D208. 10.1093/nar/gkv1252 26586799 PMC4702886

