# Supplementary figures and images for: Identification and Analysis of Long Non-coding RNAs in Leuciscus waleckii Adapted to Highly Alkaline Conditions
Source: Front Physiol. 2021 Jun 11;12:665268. doi: 10.3389/fphys.2021.665268 (PMC8232936; doi:10.3389/fphys.2021.665268)

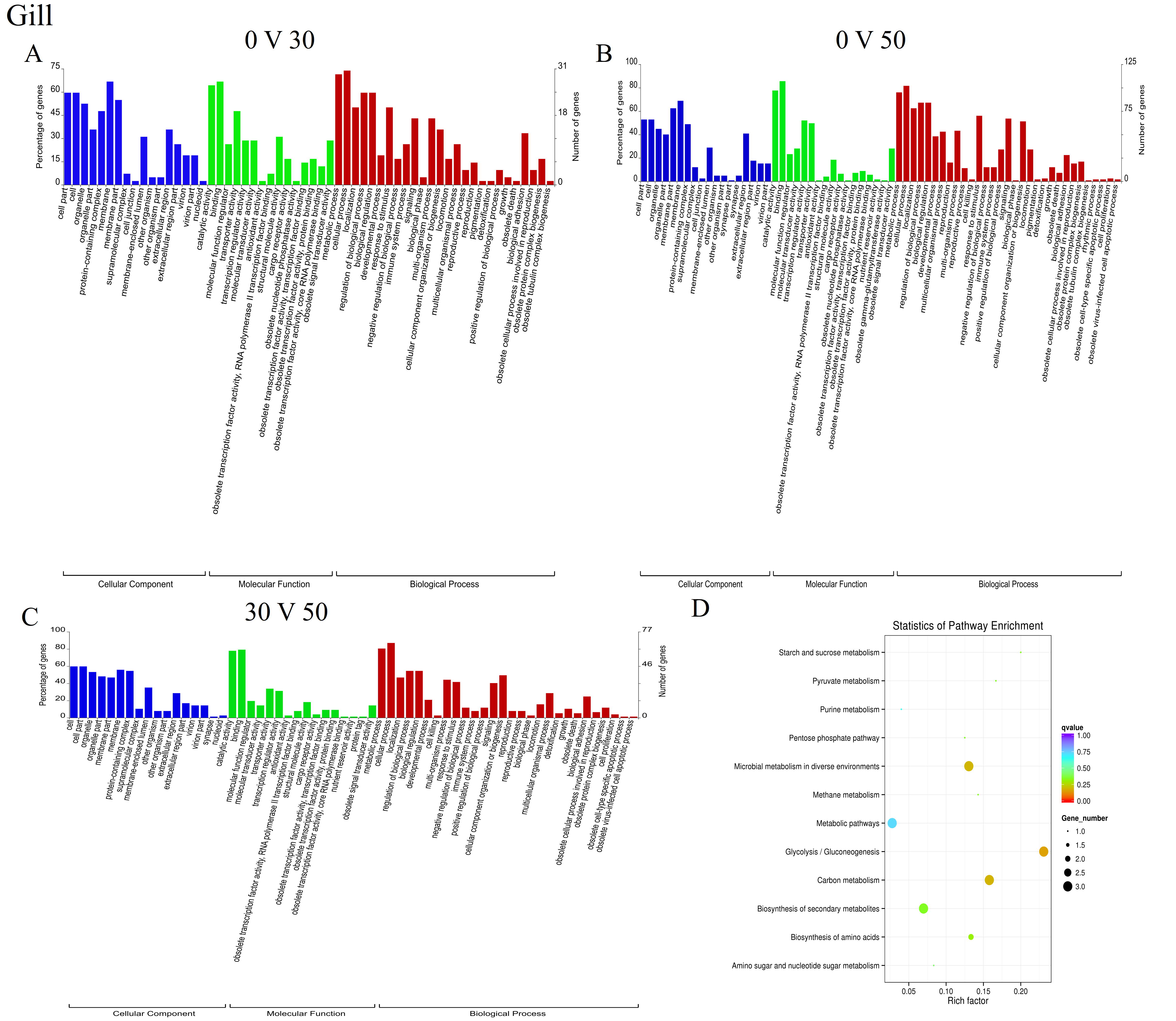

Supplement: Supplementary file 1 [file Data_Sheet_1.ZIP › supplementary material presentation/Supplementary Figure.1.jpg]

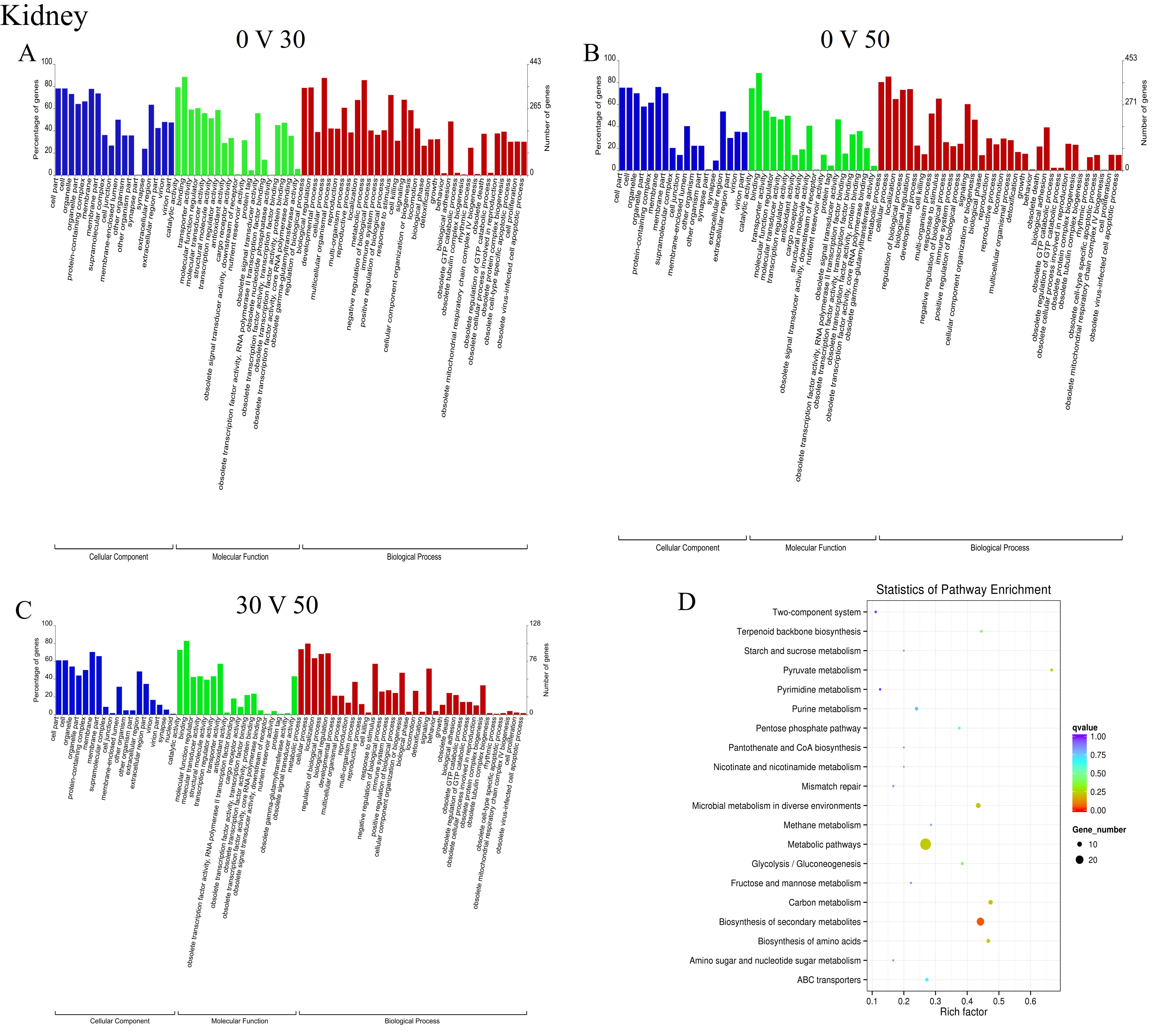

Supplement: Supplementary file 1 [file Data_Sheet_1.ZIP › supplementary material presentation/Supplementary Figure.2.jpg]
