# Supplementary material for: Identification and Analysis of Long Non-coding RNAs in Leuciscus waleckii Adapted to Highly Alkaline Conditions
Source: Front Physiol. 2021 Jun 11;12:665268. doi: 10.3389/fphys.2021.665268 (PMC8232936; doi:10.3389/fphys.2021.665268)
Supplement: Supplementary file 2 [file Data_Sheet_2.ZIP › abbreviation table/abbreviation table.pdf]

Identification and Analysis of Long Non-Coding RNAs in *Leuciscus waleckii* Adapted to Highly Alkaline Conditions

Abbreviation	Full name
lncRNA	Long Non-Coding RNAs
RT-qPCR	Quantitative real-time PCR
GO	Gene Ontology
KEGG	Kyoto Encyclopedia of Genes and Genomes
lincRNAs	Large intergenic noncoding RNAs
ANO1	Anoctamin-1
ARRDC4	Arrestin domain-containing protein 4
RUFY2	RUN, and FYVE domain-containing protein 2
KCNJ1	ATP-sensitive inward rectifier potassium channel 1
KCNJ5	G protein-activated inward rectifier potassium channel 4
atp1a1	Sodium/potassium-transporting ATPase subunit alpha-1
CALM2-B	Calmodulin-2B
RHCG1	Ammonium transporter Rh type C 1
SLC4A4	Electrogenic sodium bicarbonate cotransporter 1
SLC26A3	Chlorine anion exchanger
Slc26a6	Solute carrier family 26 member 6
CA-VB	Carbonic anhydrase 5B
SLC38A3	Sodium-coupled neutral amino acid transporter 3
NPPA	Natriuretic peptides A
CLCN6	Chloride transport protein 6
CC	Cellular component
MF	Molecular function
BP	Biological process
CLIC4	Chloride intracellular channel protein 4
CA	Carbon anhydrase
