# Supplementary material for: Identification and Analysis of Long Non-coding RNAs in Leuciscus waleckii Adapted to Highly Alkaline Conditions
Source: Front Physiol. 2021 Jun 11;12:665268. doi: 10.3389/fphys.2021.665268 (PMC8232936; doi:10.3389/fphys.2021.665268)
Supplement: Supplementary file 2 [file Data_Sheet_2.ZIP › abbreviation table/abbreviation table.doc]

**Identification and Analysis of Long Non-Coding RNAs in *Leuciscus waleckii* Adapted to Highly Alkaline Conditions**

| Abbreviation | Full name |
| --- | --- |
| lncRNA | Long Non-Coding RNAs |
| RT-qPCR | Quantitative real-time PCR |
| GO | Gene Ontology |
| KEGG | Kyoto Encyclopedia of Genes and Genomes |
| lincRNAs | Large intergenic noncoding RNAs |
| ANO1 | Anoctamin-1 |
| ARRDC4 | Arrestin domain-containing protein 4 |
| RUFY2 | RUN, and FYVE domain-containing protein 2 |
| KCNJ1 | ATP-sensitive inward rectifier potassium channel 1 |
| KCNJ5 | G protein-activated inward rectifier potassium channel 4 |
| atp1a1 | Sodium/potassium-transporting ATPase subunit alpha-1 |
| CALM2-B | Calmodulin-2B |
| RHCG1 | Ammonium transporter Rh type C 1 |
| SLC4A4 | Electrogenic sodium bicarbonate cotransporter 1 |
| SLC26A3 | Chlorine anion exchanger |
| Slc26a6 | Solute carrier family 26 member 6 |
| CA-VB | Carbonic anhydrase 5B |
| SLC38A3 | Sodium-coupled neutral amino acid transporter 3 |
| NPPA | Natriuretic peptides A |
| CLCN6 | Chloride transport protein 6 |
| CC | Cellular component |
| MF | Molecular function |
| BP | Biological process |
| CLIC4 | Chloride intracellular channel protein 4 |
| CA | Carbon anhydrase |
